# Wavelet LSTM for Fault Forecasting in Electrical Power Grids

**DOI:** 10.3390/s22218323

**Published:** 2022-10-30

**Authors:** Nathielle Waldrigues Branco, Mariana Santos Matos Cavalca, Stefano Frizzo Stefenon, Valderi Reis Quietinho Leithardt

**Affiliations:** 1 Department of Electrical Engineering, Santa Catarina State University, R. Paulo Malschitzki 200, Joinville 89219-710, Brazil; 2 Fondazione Bruno Kessler, Via Sommarive 18, 38123 Trento, Italy; 3 Department of Mathematics, Informatics and Physical Sciences, University of Udine, Via delle Scienze 206, 33100 Udine, Italy; 4 COPELABS, Lusófona University of Humanities and Technologies, Campo Grande 376, 1749-024 Lisboa, Portugal; 5 VALORIZA, Research Center for Endogenous Resources Valorization, Instituto Politécnico de Portalegre, 7300-555 Portalegre, Portugal

**Keywords:** electrical power grids, fault forecasting, long short-term memory, time series forecasting, wavelet transform

## Abstract

An electric power distribution utility is responsible for providing energy to consumers in a continuous and stable way. Failures in the electrical power system reduce the reliability indexes of the grid, directly harming its performance. For this reason, there is a need for failure prediction to reestablish power in the shortest possible time. Considering an evaluation of the number of failures over time, this paper proposes performing failure prediction during the first year of the pandemic in Brazil (2020) to verify the feasibility of using time series forecasting models for fault prediction. The long short-term memory (LSTM) model will be evaluated to obtain a forecast result that an electric power utility can use to organize maintenance teams. The wavelet transform has shown itself to be promising in improving the predictive ability of LSTM, making the wavelet LSTM model suitable for the study at hand. The assessments show that the proposed approach has better results regarding the error in prediction and has robustness when statistical analysis is performed.

## 1. Introduction

For electricity to reach consumers in a stable and continuous way, an electrical power grid must work independently of weather conditions [1]. To keep the electrical distribution system running, it is necessary to evaluate the performance of the electrical system’s equipment through simulation, and then the disturbance conditions present in the electrical power grid can be identified [2]. Disturbances that occur in an electrical power system can significantly affect the power supply, variations in voltage level, and increased surface conductivity, or contact by the conductors with the ground can result in faults, which affect power quality [3].

Time series forecasting can be used to identify the possibility of a failure occurring, which is a promising way to assist the decision-making process for maintenance teams in an electric power utility [4]. As the increase in failures has a strong relationship with weather conditions, in rainy seasons, there is a greater chance of a failure occurring, so the study of this variation in relation to a time series is an important aspect in this context [5].

The use of wavelet transforms for noise reduction is an approach that is effective when there is high nonlinearity in the time series [6]. When using high-frequency bandwidth filters, there may be a loss of information, considering that a high frequency might be related to the occurrence of a failure. Considering that the wavelet transform evaluates the signal energy, high frequencies are not totally eliminated, thus maintaining the main signal characteristics [7]. Thus, a hybrid approach that combines a deep learning model with a wavelet transform can be an interesting approach [8].

Long short-term memory (LSTM) is a model applied in deep learning that has been widely used by researchers for time series forecasting [9,10,11]. Its units solve the vanishing gradient problem partially, since LSTM units allow the gradients to flow unchanged [12]. Based on the advantages of the wavelet transform and the promising capabilities of LSTM [13,14,15,16], this work proposes using a combination of those techniques in a method named wavelet LSTM. For this purpose, a study will be conducted using the alarm data obtained from a recloser of a power utility company in the *Serrana* region of Santa Catarina, Brazil.

The main contributions of this research are the following:

- We propose a hybrid wavelet LSTM model which has a higher predictive capacity than the standard LSTM model. The wavelet LSTM shows itself to be a more stable model for time series prediction that can be used in several applications.
- We evaluate a time series regarding the variation in the number of failures in distribution networks with bare cables due to the presence of contamination and contact of foreign materials with the grid, resulting in disruptive discharges in the power grid.
- We present a solution for evaluating failure history based on a time series that can be used in other works, in which it is necessary to evaluate the number of failures over time.

The continuation of this paper is organized as follows. Section 2 presents a review of related works and the used data. Section 3 presents the proposed method. In Section 4, the results are analyzed. Section 5 presents a conclusion and a discussion of possible future works.

## 2. Related Works

In electrical distribution systems, an electrical fault is defined as an anomaly in a particular piece of equipment causing a forced interruption in the operation of the electrical power grid [17]. There are two classes of faults: transient and permanent. Transient faults are anomalies of short duration that disappear soon after the action of protective devices, having as common causes atmospheric discharges, momentary contacts between the conductors and the ground, the opening of an electric arc, and materials without adequate insulation. Permanent faults are faults that continue to exist until it is possible to replace the defective component or equipment [18].

Through fault diagnosis, it is possible to detect where the fault occurred as well as its size, duration, and impact on the electrical power system [19]. Among the most current fault diagnosis methods are Bayesian networks [20], fuzzy logic [21], the Kalman filter [22], and other mathematical models based on artificial intelligence. The use of artificial intelligence techniques for fault identification has been growing over the years and become a hot topic today, especially for electric power systems [23]. Deep learning models have been increasingly used to improve the ability to identify faults in an electrical grid [24,25,26]. However, as these models have a large number of layers, they require more computational effort, making the choice of the appropriate model a challenge [27].

From the image processing of failed components, it is possible to identify patterns and thus improve their identification in the field [28]. Several researchers are using object detection and image classification based on convolutional neural network (CNN) models [29,30,31].

CNNs can be specifically applied to improve the ability to identify faulty components, as shown by Liu et al. [32] and Sadykova et al. [33] using the You Only Look Once (YOLO) approach, Li et al. [34] with an improved Faster R-CNN, and Wen et al. [35] using an Exact R-CNN. As presented by Sadykova et al. [33], the YOLO model is a promising alternative for identifying insulators during power grid inspections and handling large datasets, where data augmentation techniques can also be applied to avoid early overfitting. In this context, a super-resolution CNN can perform reconstruction of the blurred images to perform the expansion of the dataset [36].

The YOLO model has been updated, and variations in its structure can result in significant performance improvements. According to Liu et al. [32], the YOLOv3-dense model proposed by them reached up to 94.47% for insulator identification using varied image backgrounds. In comparison, for the same dataset, YOLOv3 reached 90.31% accuracy. Previous versions such as YOLOv2 reached a maximum of 83.43%, making it clear that in many situations, the use of non-standard models can be a promising alternative.

Variations of the YOLO models have been proven to be very efficient for locating insulators on transmission lines. According to Liu et al. [37], MTI-YOLO has a higher average precision than YOLO-tiny and YOLO-v2. Liu et al. [38] proposed an improved YOLOv3 model that is better than the YOLOv3 and YOLOv3-dense models. Hu and Zhou [39] showed that YOLOv4 can reach an accuracy of 96.2% for insulator defect detection, and using YOLOv4, Xing and Chen [40] had a precision of 97.78% for insulator identification.

When the distribution power system does not have insulation on the medium voltage conductors, trees might touch the conductors, resulting in discharges to the ground [41]. This type of fault is common in rural power grids that are close to wooded areas. To prevent these faults, electric power utilities perform pruning of trees that are close to the network, thus reducing the chance of discharges to the ground [42].

Insulators installed outdoors are exposed to environmental variations, such as dust accumulation on their surfaces [43]. When contamination accumulates on insulators, their surface conductivity increases, generating leakage current until a discharge occurs [44]. When there is high humidity in the air, the conductivity increases even more, consequently increasing the chance of faults in the grid [45]. One type of contamination that has a significant impact on the conductivity of insulators is salt contamination, which can be measured by the equivalent salt deposit density [46].

Considering all these possible faults [47], time series forecasting comes as an alternative to prepare maintenance teams in advance for an event based on the historical knowledge of data variation over time [48]. A forecast that is many steps ahead is challenging, as each step ahead contains the accumulated forecast error of the previous step [49]. Therefore, time series forecasting needs to take into account how many steps ahead can be considered to obtain acceptable assertiveness [50].

Among the algorithms for time series forecasting, ensemble learning models in general have high performance and lower computational effort [51], and they may be promising approaches for failure prediction. Various ways of combining the weak learners can be used to create a model that has a greater capacity, such as bagging, boosting, and stacking [52]. Further optimized models, such as the Bayesian optimization-based dynamic ensemble proposed by Du et al. [53], can be used and are even applied with nonlinear data [54].

Many variations of ensemble models for time series forecasting can be found, such as efficient bootstrap stacking, presented by Ribeiro et al. [55], or extreme gradient boosting, proposed by Sauer et al. [56]. Especially for power system failure prediction, the wavelet transform combined with ensemble models becomes a superior approach to well-established models, such as the adaptive neuro-fuzzy inference system [57]. Therefore, ensemble models are successful approaches for multi-step forward prediction [58], which is equivalent to what is being evaluated in this paper.

Due to the existing features in the structure of LSTM, it is one of the most qualified models for handling chaotic time series, since it has the ability to remember distant values and interpret the order of dependencies, which are essential characteristics for prediction models. Abbasimehr and Paki [59] used the attention mechanism to attain an enhanced LSTM model. Related to the power system using LSTM, Guo et al. [60] and Ko et al. [61] presented research about wind power forecasting. Specifically for fault prediction, Guo et al. [62] proposed a modified LSTM version to improve the safe and reliable operation of mechanical equipment.

### Faults

Most faults that occur in an electrical power distribution system with naked cable are caused by direct contact with the network. This occurs mainly when the weather conditions are bad (rain and intense wind), increasing the likelihood of contact from trees with the power grid. Another failure that can occur frequently is when insulators lose their insulating capacity due to contamination or when the insulators are damaged [63].

To perform time series evaluation, all failures that occur on the same day are added up to obtain a daily failure rate over time and thus evaluate the influence of the change in season in relation to the increase in failures in an electrical power grid. These failures are evaluated in relation to the alarms registered by the electric power utility company during the evaluated period. Some examples of alarms are presented in Table 1.

The alarms listed in Table 1 contain the day, time, and reason for the failure. The original dataset, which presents all recorded alarms, is available at https://github.com/SFStefenon/FailuresPowerGrid2020 (accessed on 21 October 2021).

Since failures generally occur in a nonlinear pattern, this evaluation was based on statistical analysis, and it was not possible to determine exactly when a failure would occur. However, it was possible to evaluate in which period of the year there was a greater chance of the highest number of failures occurring.

**Figure 1:**
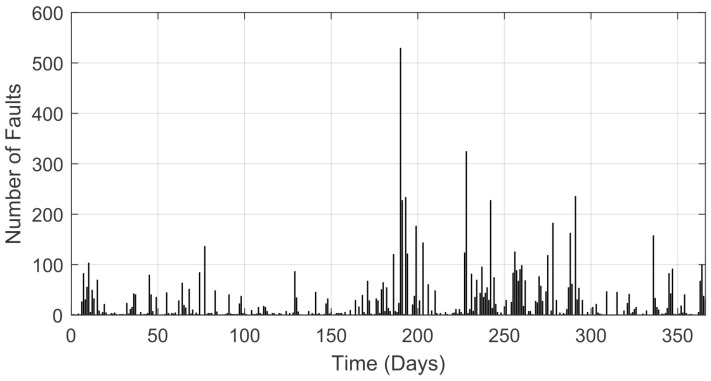
Failures registered in the power grid in 2020 (Lages region).

In this paper, the evaluation of the history of recorded faults is in relation to the year 2020 (from 1 January to 31 December), and this history corresponds to the sum of all the faults of the distribution branches in the Lages region (Brazil) based on data provided by *Centrais Elétricas de Santa Catarina* (CELESC). In total, there were 366 days recorded, considering that the year 2020 was a leap year. Figure 1 presents the sum of the alarms regarding faults per day in this period.

## 3. Wavelet LSTM

The wavelet LSTM method is a combination of the wavelet transform and long short-term memory. This approach has been widely used for fault diagnosis, as presented in the work of Sabir et al. [64] and Jalayer, Orsenigo, and Vercellis [65] for electrical machines, and especially for rolling bearings in the work of Tan et al. [66]. In scenarios that involve time series forecasting, its application can be extended to the Internet of Things [67,68,69], industry applications [70,71,72], and sustainability [73].

**Figure 2:**
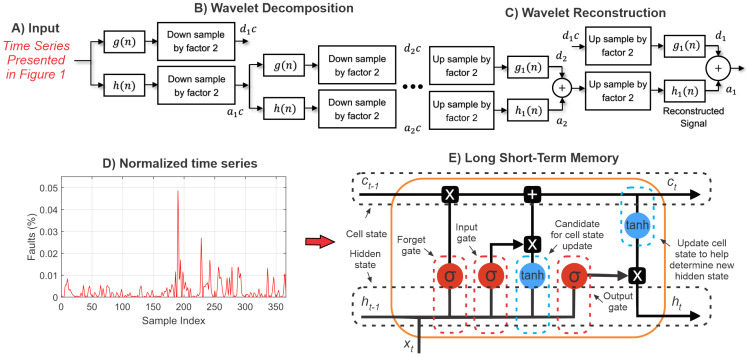
Structure of the wavelet long short-term memory model.

To apply the wavelet LSTM model here, initially, the time series passes through the wavelet filter to reduce the noise and nonlinearities. After the signal is decomposed and reconstructed, the LSTM receives the filtered signal and performs the prediction. The complete structure of this approach is presented in Figure 2 and will be explained in this section.

The structure of the proposed method (presented in Figure 2) can be divided into steps as follows. In the first step (A), the original input signal (shown in Figure 1) is loaded. In the next step, the wavelet transform is applied, which is divided into two parts, namely the signal decomposition (B) and its reconstruction (C) into the time series. In the next step, the denoising signal is normalized (D), where the variation in the number of failures is evaluated in relation to all recorded faults in the considered period. In the last step, the LSTM model is used to perform the time series prediction one step ahead (E).

To use the wavelet transform, the signal was first decomposed using the wavelet packet transform (WPT) method to obtain the energy coefficient of the signal. This procedure considers both sides of the spectrum (high and low frequencies). The decomposition may be denoted by

(1) $$W_{\Psi ,x}\left(A,B\right)=\frac{1}{\sqrt{A}}\int_{-\infty }^{+\infty }x\left(t\right)\Psi *\left(\frac{t-B}{A}\right)dt,\;A\neq 0\;$$

where x(t) is the signal to be decomposed, Ψ is the time-based function (mother wavelet), and *A* and *B* are the scale and displacement parameters, respectively [74]. Given a discretization, the high-pass filter g(n) is

(2) $$g\left(n\right)=h\left(2N-1-n\right).$$

where h(n) is the low-pass filter. Thereby, the mother wavelet and the scaling function (Φ) are given by

(3) $$\Psi \left(n\right)=\sum_{i=0}^{N-1}g\left(i\right)\Phi \left(2n-i\right),$$

(4) $$\Phi \left(n\right)=\sum_{i=0}^{N-1}h\left(i\right)\Phi \left(2n-i\right).$$

**Figure 3:**
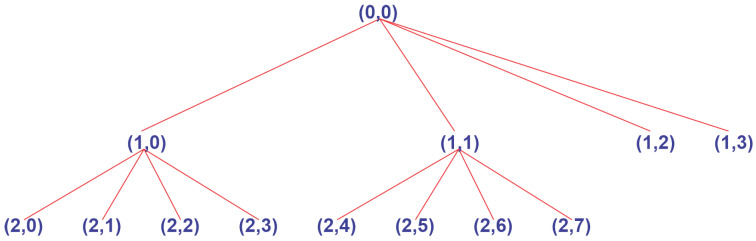
Tree decomposition.

The WPT performs a new decomposition on each interaction using the coefficients from the previous iterations and therefore indicates that the total number of coefficients is determined by the number of iterations. Each wavelet packet coefficient can be determined based on its frequency level. The WPT decomposes the all elements of the frequencies, and thus its use results in both low- and high-frequency components. By using the tree structure created by the approximation decomposition coefficients, an optimal binary value is obtained. An example of the tree structure for wavelet decomposition is presented in Figure 3.

As can be seen, paths 1,2 and 1,3 are not used after optimizing the structure, resulting in an optimized decomposition. After wavelet packet decomposition based on the optimum binary wavelet packet tree, the signal is reconstructed while considering the number of defined nodes. With the reconstructed filtered signal, a time series is obtained that is used for LSTM forecast evaluation.

LSTM is a recurrent neural network that has feedback, allowing the model to remember distant values. For the time series forecasting starting from *D* samples, we use

(5) x(t−(D−1)Δ),…,x(t−Δ),x(t)

to predict the future value

(6) x(t+P),

where *P* represents the steps forward and Δ is the period of the samples. In this paper, Δ is equal to 1 day, where all faults of the same day were summed. In this paper, one-step-ahead prediction was used (*P* = 1).

LSTM is capable of understanding order dependence in problems that require sequence prediction, making it promising for time series forecasting [75]. In an LSTM algorithm, each cell is divided into three gates: the input ($\iota_{t}$), output ($o_{t}$), and forgetting ($f_{t}$) gates [76], where $f_{t}$ controls how much information will be forgotten and how much will be remembered, and the useful information for the states is added through the $\iota_{t}$ and $o_{t}$ to determine how much of the current state must be assigned to the output [77]. The LSTM can be defined by the following equations:

(7) $$\begin{array}{c} \iota_{t}=\sigma_{g}\left(W_{\iota }x_{t}+R_{\iota }h_{t-1}+b_{\iota }\right),\\ f_{t}=\sigma_{g}\left(W_{f}x_{t}+R_{f}h_{t-1}+b_{f}\right),\\ o_{t}=\sigma_{g}\left(W_{o}x_{t}+R_{o}h_{t-1}+b_{o}\right). \end{array}$$

where *b* is the polarization matrix, *R* and *W* are earnings matrices, and $\sigma_{g}$ is the activation function [78]. LSTM has an input activation function *G* and output activation function *H*, which are used to update the cell and the hidden state as given in the following equations:

(8) $$\begin{array}{c} {\tilde{c}}_{t}=G\left(W_{c}x_{t}+R_{c}h_{t-1}+b_{c}\right),\\ c_{t}=f_{t}\circ c_{t-1}+\iota_{t}\circ \tilde{c},\\ h_{t}=o_{t}\circ H\left(c_{t}\right). \end{array}$$

To find the predicted values of future time steps, the training response sequences are shifted by a time step. Thus, at each time step in the input sequence, the net learns to forecast the following time-step value. To feed the compared models, normalization is performed using the values as percentages in relation to the total number of failures recorded in the evaluated period.

To improve the efficiency of the proposed model, three optimizers were evaluated. Stochastic gradient descent (SGD) updates the parameters of the neural net to minimize the loss function by taking small steps in each iteration (*i*) in the direction of the negative loss gradient:

(9) $$\theta_{i+1}=\theta_{i}-\alpha \nabla E\left(\theta_{i}\right).$$

SGD with momentum (SGDM) helps accelerate the gradient vectors in the correct directions, resulting in faster convergence [79]. Here, α is the learning rate, θ is the vector of parameters, and $E(\theta_{i})$ is the loss function to be optimized.

Root mean squared propagation (RMSProp) employs learning rates that differ per parameter and can adapt automatically to the loss function that is optimized. In this way, the algorithm keeps a moving average of the squares of the parameter, which is calculated as follows:

(10) $$v_{i}=\beta_{2}v_{i-1}+\left(1-\beta_{2}\right){ [\nabla f(x_{i})]}^{2}.$$

where $\beta_{2}$ is the decay rate of the moving average. The algorithm takes the moving average to normalize the parameter updates individually [80]:

(11) $$x_{i+1}=x_{i}-\frac{\alpha \nabla f(x_{i})}{\sqrt{v_{i}}+\varepsilon }\;.$$

The adaptive moment estimation (ADAM) method computes the learning rates for each parameter. The downward averages of the last $m_{i}$ and the squared gradients of the last $v_{i}$ are calculated as follows:

(12) $$m_{i}={\beta_{1}m}_{i-1}+\left(1-\beta_{1}\right)\nabla f\left(x_{i}\right),$$

(13) $$v_{i}={\beta_{2}m}_{i-1}+\left(1-\beta_{2}\right){ [\nabla f(x_{i})]}^{2}.$$

ADAM works by using moving averages to update the parameters of the network in the following way:

(14) $$x_{i+1}=x_{i}-\frac{\alpha m_{1}}{\sqrt{v_{i}}+\varepsilon }.$$

### Considered Measures

In this paper, the root mean square error (RMSE), mean absolute error (MAE), and coefficient of determination (R${}^{2}$) were considered, given by

(15) $$\mathrm{RMSE}=\sqrt{\frac{1}{\eta }\sum_{i=1}^{\eta }{\left(y_{i}-{\hat{y}}_{i}\right)}^{2}},$$

(16) $$\mathrm{MAE}=\frac{1}{\eta }\sum_{i=1}^{\eta }\left|y_{i}-{\hat{y}}_{i}\right|,$$

(17) $$R^{2}=1-\frac{\sum_{i=1}^{\eta }{\left(y_{i}-{\hat{y}}_{i}\right)}^{2}}{\sum_{i=1}^{\eta }{\left(y_{i}-{\bar{y}}_{i}\right)}^{2}},$$

where $y_{i}$ is the observed value, ${\hat{y}}_{i}$ is the predicted output, and ${\bar{y}}_{i}$ is the average of the observed value [81].

The statistical evaluation was performed with 50 runs using the same parameter configuration, where the mean, median, and standard deviation were evaluated. The simulations were computed using an Intel Core I5-7400 with 20 GB of RAM using MATLAB software. For a comparative assessment, the adaptive neuro-fuzzy inference system (ANFIS) [82,83], group method of data handling (GMDH) [84], bagging [85], random subspace [86], and stacking [87] ensemble learning methods were evaluated.

## 4. Analysis of Results

The first evaluation regarded the analysis of the time series forecast performed in relation to the percentage of data used for training and testing of the neural network. The evaluation of this parameter is important because it can be used to define the minimum amount of data needed for training the model. The best results presented in this section are highlighted in bold.

The evaluation results are shown in Table 2, considering a training ratio from 50 to 90 percent. The test used the difference equivalent percentage to complete the dataset, in which the validation stage was not considered.

**Figure 4:**
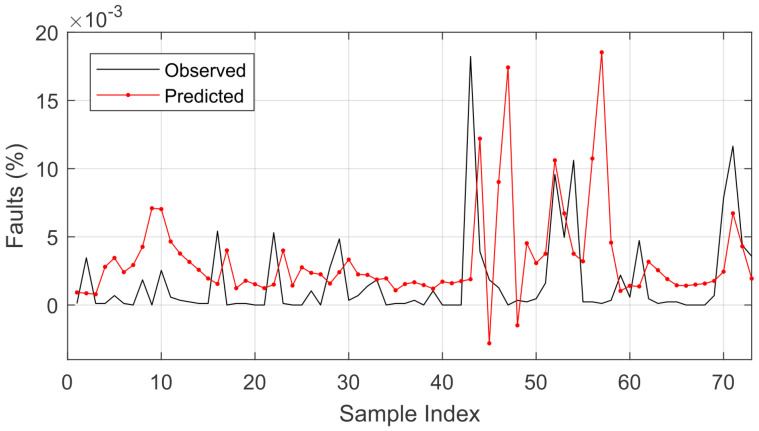
Preliminary analysis of fault prediction capability.

Using 80% of the data for training and 20% of the data for testing gave the best RMSE and R${}^{2}$ values, and hence this ratio was used in further analysis. As can be seen in Figure 4, there was major difficulty in predicting the data due to the nonlinearities in the time series, considering that in some cases, there were several failures in a short period of time.

The failures that occurred after the middle of the year were due to the rainy season, which starts after winter in the Southern Hemisphere. The greater presence of bad weather conditions favors the development of faults in electrical power distribution systems. In the following analysis, the optimizer and the number of hidden units are evaluated (see Table 3).

In this evaluation, the SGDM optimizer had more stable results for the coefficient of determination, presenting a smaller variance in relation to the change in hidden units. When comparing all the models, the best results occurred using 200 hidden units, considering the coefficient of determination. In some cases, it was not possible to measure the coefficient of determination due to the high intensity of the variation in the prediction using the ADAM and RMSprop optimizers. Considering that there was a large variation in the values, statistical analysis was performed (and will be presented) using 200 hidden units.

**Figure 5:**
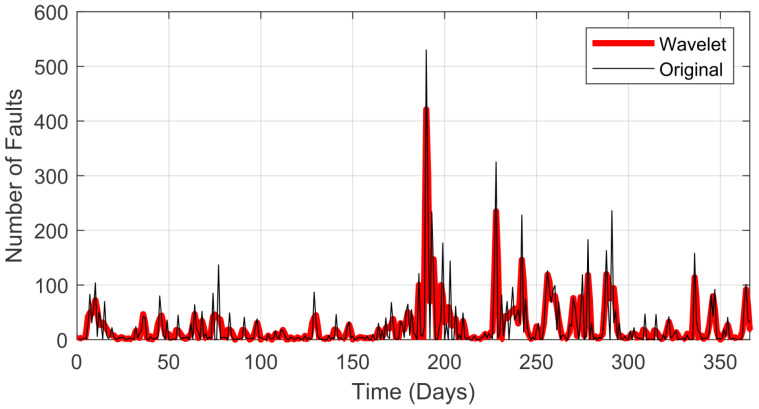
Evaluation of the wavelet transform with one node.

**Figure 6:**
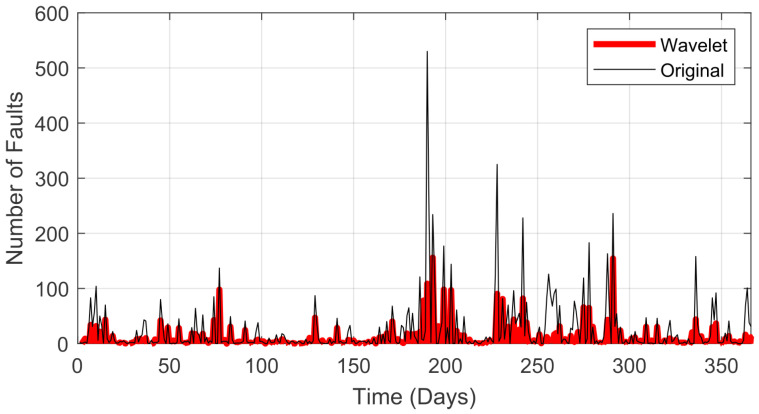
Evaluation of the wavelet transform with two nodes.

A wavelet transform for noise reduction was added for the following analysis. This transform should be used with caution, as it can result in a loss of features for the signal. Figure 5 shows the results of the wavelet transform relative to the original signal using one node, and Figure 6 shows this comparison using two nodes.

The use of two nodes considerably altered the response of the transform, hindering practical application. When three nodes or more are used, the signal loses its characteristics, and this was not considered in this paper.

The complete analysis of the wavelet transform depth variation is presented in Table 4. Considering that there was an error value that made the prediction not suitable for analysis, the use of two nodes was disregarded after this evaluation. As mentioned earlier this can also be observed when the wavelet transform was compared in this configuration in relation to the original signal (see Figure 6).

The best coefficient of determination was reached using a depth equal to two in the wavelet transform, getting close to the best MAE value, which happened using a depth equal to four. Considering these results, a depth equal to two was used for statistical analysis, which is presented in Table 5.

**Figure 7:**
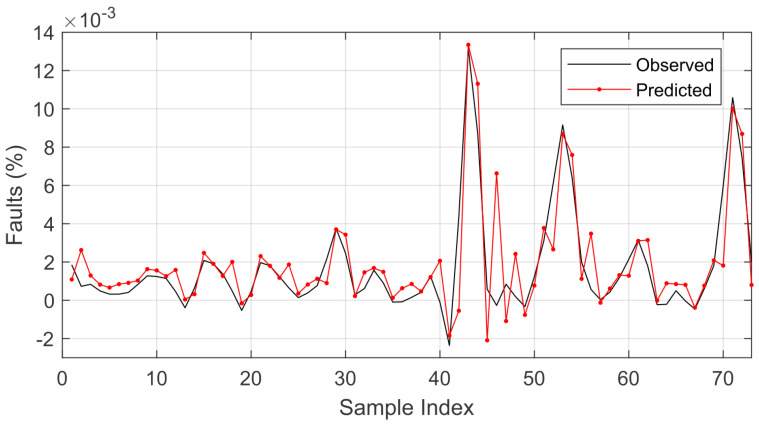
Comparison of the prediction using the wavelet LSTM model for the observed values.

The wavelet LSTM model was superior in all comparative analyses to the LSTM model with respect to the RMSE. Even when varying the optimizer, the wavelet LSTM model showed promise for the analysis in question. The best average RMSE result was obtained using the RMSprop optimizer in the wavelet LSTM model. The comparison between the prediction result and the original signal is presented in Figure 7.

The results of the comparison of the predicted and observed values (shown in Figure 7) mean that it would be possible to estimate the next day’s failures based on the recorded history (considering that the prediction is one step ahead, which corresponds to 1 day). As the forecast is accomplished one step ahead, the history of the data recorded until the next expected forecast is used to predict the next one. Considering that the forecast is performed in relation to the sum of the failure records that occurred over time, it would be possible to estimate the number of failures for the next day.

Whereas failures are related to weather conditions, there is a tendency for them to increase depending on the time of year, which is the focus of this research. After the highest accumulated value of the number of failures, there was an oscillation in the prediction, something that was expected due to this abrupt variation in the time series.

**Figure 8:**
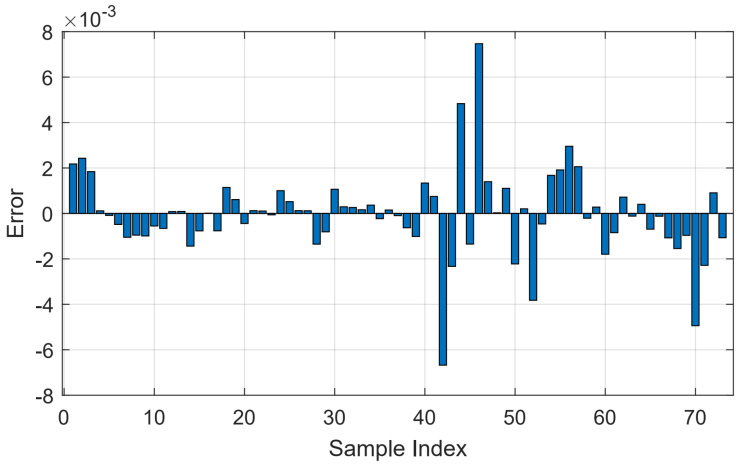
Error given by the difference between predicted and observed values.

This statement is supported based on the error calculated by the difference between the predicted and observed values, as presented in Figure 8.

Once the best parameter configurations for the LSTM wavelet model were defined, benchmarking, presented in Table 6, was performed, aiming to compare the proposed model with well-established models.

The LSTM wavelet model presented the best results regarding the RMSE and MAE, despite requiring the longest time to converge. The ensemble stacking model presented the best coefficient of determination with the shortest time required for convergence of the ensemble models, with this being an indication that this model may be promising in this evaluation. The other models compared presented similar RMSE results, not being superior to the proposed model. The ANFIS model using the radius of influence did not converge because the time series used had insufficient data for this structure.

## 5. Conclusions

The prediction of faults in an electrical distribution system is necessary to ensure the operation of the power grid. By analyzing the variation of a time series, it is possible to verify the presence of a higher number of failures during a certain time and thus define a more effective correction strategy. Based on time series forecasting, an electric utility company may know when there are higher chances to have faults before they happen and thus have more defined strategies to deal with them.

It is noticeable that there was difficulty in this prediction due to the large variation in the number of failures in some seasons of the year, which was mainly related to the rainy season. Using traditional models, the forecast results were ineffective, so it was necessary to combine algorithms and create a hybrid model to meet the needs of the problem.

The wavelet LSTM model showed better results in all analyses compared with the standard LSTM model, including better results in the statistical analysis, being an appropriate model for the evaluation presented in this paper. Using this model, it is possible to have failure prediction indicators that can help the organization of maintenance teams, thus reducing the response time when a disruptive failure occurs.

The LSTM model has a high predictive ability for chaotic time series, which was the case in the need for solving this task. Using the LSTM model without the inclusion of additional filters made it impossible to perform an acceptable prediction, considering that the abrupt variation in the time series of the number of failures made it necessary to perform a smoothing of the series through the inclusion of a filter. The results showed that the wavelet LSTM model was acceptable for the analysis in question, being superior to the ensemble learning methods, GMDH, and ANFIS.

Future work can be conducted regarding the type of failure. The failures can vary, for instance, because of direct contact with the grid or leakage current. Specific analysis on which type of failure occurs more frequently and how to avoid this type of failure is promising work to be carried out in the future.

## Figures and Tables

**Table 1:** Example of alarms that have been registered in the considered period.

Day	Time	Failure Record
6 January 2020	11:09:41	Current Phase B
6 January 2020	11:09:50	Current Phase A
6 January 2020	17:10:32	Current Phase C
26 January 2020	13:57:37	Recloser Communic. Failure
06 April 2020	10:04:23	Relay 50/51 (Neutral)
01 June 2020	17:24:51	Current Phase A
30 June 2020	14:11:56	Phase Voltage C
27 August 2020	10:00:54	Neutral Protection
27 August 2020	11:58:48	Current Phase C
11 September 2020	03:06:12	Current Phase A
29 December 2020	13:56:32	Relay 50/51 (Phase A)

**Table 2:** Evaluating the influence of the training and testing relationship.

Train/Test	RMSE	MAE	R${}^{2}$	Time (s)
50/50	8.02 × 10${}^{-3}$	3.03 × 10${}^{-3}$	0.1516	**17.21**
60/40	6.24 × 10${}^{-3}$	7.47 × 10${}^{-4}$	**0.1681**	18.53
70/30	4.73 × 10${}^{-3}$	3.59 × 10${}^{-4}$	0.0325	18.64
80/20	**3.60 × 10${}^{-3}$**	1.18× 10${}^{-3}$	0.2779	20.47
90/10	4.29 × 10${}^{-3}$	**5.49 × 10${}^{-5}$**	0.0884	19.68

**Table 3:** Assessment of the number of hidden units (HUs) using different optimizers.

Optimizer	HU	RMSE	MAE	R${}^{2}$	Time (s)
	50	3.49 × 10${}^{-3}$	9.12 × 10${}^{-4}$	0.1957	**17.74**
	100	3.50 × 10${}^{-3}$	9.12 × 10${}^{-4}$	0.2035	18.29
SGDM	200	3.51 × 10${}^{-3}$	1.17 × 10${}^{-3}$	0.2089	19.58
	500	3.44 × 10${}^{-3}$	9.18 × 10${}^{-4}$	0.1624	25.06
	1000	3.44 × 10${}^{-3}$	9.37 × 10${}^{-4}$	0.1623	36.66
	50	4.79 × 10${}^{-3}$	1.77 × 10${}^{-3}$	-	17.94
	100	7.69 × 10${}^{-3}$	4.58 × 10${}^{-3}$	-	19.94
ADAM	200	3.98 × 10${}^{-3}$	6.51 × 10${}^{-4}$	**0.5554**	19.21
	500	3.96 × 10${}^{-3}$	8.29 × 10${}^{-4}$	0.5469	25.55
	1000	3.94 × 10${}^{-3}$	1.52 × 10${}^{-3}$	0.5242	35.22
	50	6.22 × 10${}^{-3}$	3.01 × 10${}^{-3}$	-	21.58
	100	5.65 × 10${}^{-3}$	1.22 × 10${}^{-3}$	-	19.14
RMSprop	200	3.93 × 10${}^{-3}$	6.64 × 10${}^{-4}$	0.5212	21.64
	500	3.35 × 10${}^{-3}$	1.00 × 10${}^{-3}$	0.1074	27.51
	1000	**3.16 × 10${}^{-3}$**	**2.96 × 10${}^{-5}$**	0.0190	36.50

**Table 4:** Assessment of depth using different one and two nodes.

Nodes	Depth	RMSE	MAE	R${}^{2}$	Time (s)
	1	2.19 × 10${}^{-3}$	4.54 × 10${}^{-4}$	0.3375	23.14
	2	**2.16 × 10${}^{-3}$**	4.69 × 10${}^{-4}$	0.3547	22.13
1	3	2.22 × 10${}^{-3}$	5.28 × 10${}^{-4}$	0.3132	23.61
	4	**2.16 × 10${}^{-3}$**	**3.53 × 10${}^{-4}$**	0.3509	22.76
	5	2.17 × 10${}^{-3}$	3.70 × 10${}^{-4}$	0.3493	21.71
	1	2.22 × 10${}^{10}$	1.24 × 10${}^{9}$	0.6888	21.34
	2	2.13 × 10${}^{10}$	1.24 × 10${}^{9}$	**0.7155**	22.75
2	3	2.45 × 10${}^{10}$	6.79 × 10${}^{9}$	0.6214	**18.05**
	4	2.32 × 10${}^{10}$	1.90 × 10${}^{9}$	0.6601	18.74
	5	2.44 × 10${}^{10}$	6.58 × 10${}^{9}$	0.6244	18.69

**Table 5:** Statistical evaluation.

Model	Optimizer	Mean	Median	Std Dev.
	SGDM	3.50 × 10${}^{-3}$	3.49 × 10${}^{-3}$	2.62 × 10${}^{-5}$
LSTM	ADAM	7.87 × 10${}^{-3}$	8.04 × 10${}^{-3}$	2.63 × 10${}^{-3}$
	RMSprop	4.90 × 10${}^{-3}$	4.95 × 10${}^{-3}$	6.41 × 10${}^{-4}$
	SGDM	2.19 × 10${}^{-3}$	2.19 × 10${}^{-3}$	**2.76× 10${}^{-5}$**
Wavelet LSTM	ADAM	1.79 × 10${}^{-3}$	**1.27 × 10${}^{-3}$**	2.27× 10${}^{-3}$
	RMSprop	**1.67 × 10${}^{-3}$**	1.54 × 10${}^{-3}$	7.15× 10${}^{-4}$

**Table 6:** Benchmarking.

Method	Structure	RMSE	MAE	R${}^{2}$	Time (s)
	Bagging	3.33 × 10${}^{-3}$	8.55 × 10${}^{-4}$	0.0830	6.16
Ensemble	Random Subspace	3.38 × 10${}^{-3}$	1.03 × 10${}^{-3}$	0.1149	2.18
	Stacking	4.24 × 10${}^{-3}$	2.48 × 10${}^{-3}$	**0.7536**	1.17
	3 Max Layers	3.60 × 10${}^{-3}$	5.24 × 10${}^{-4}$	0.2621	**0.60**
GMDH	5 Max Layers	3.62 × 10${}^{-3}$	7.87 × 10${}^{-4}$	0.2796	1.23
	10 Max Layers	3.52 × 10${}^{-3}$	7.65 × 10${}^{-4}$	0.2088	2.52
	FCM Clustering	3.68 × 10${}^{-3}$	5.90 × 10${}^{-4}$	0.3226	4.86
ANFIS	Grid Partitioning	3.91 × 10${}^{-3}$	4.23 × 10${}^{-4}$	0.4866	12.39
	Influence Radius	-	-	-	-
Wavelet LSTM	RMSprop Opt.	**1.45 × 10${}^{-3}$**	**4.11 × 10${}^{-5}$**	0.7064	21.032

## Data Availability

The data used in this paper were provided by *Centrais Elétricas de Santa Catarina* regarding the alarms of the power distribution grids in the Lages region in Brazil from 1 January to 31 December 2020. These records are available at https://github.com/SFStefenon/FailuresPowerGrid2020 (accessed on 21 October 2021).

## Abbreviations and Symbols

The following abbreviations and symbols are used in this manuscript:

**Abbreviation**	**Meaning**
ADAM	Adaptive moment estimation
ANFIS	Adaptive neuro-fuzzy inference system
CNN	Convolutional neural network
GMDH	Group method of data handling
LSTM	Long short-term memory
MAE	Mean absolute error
RMSE	Root mean square error
RMSProp	Root mean squared propagation
SGD	Stochastic gradient descent
SGDM	Stochastic gradient descent with momentum
WPT	Wavelet packet transform
YOLO	You Only Look Once
**Symbol**	**Meaning**
*b*	Polarization matrix
*f*	Forgetting gate
*g*	High-pass filter
*i*	Iteration
*h*	Low-pass filter
*m*	Elementary moving average
*n*	Number of samples
*o*	Output gate
*t*	Time step
*v*	Moving average of the squares of the elements
*x*	Original signal
*y*	Observed value
$\hat{y}$	Predicted output
$\bar{y}$	Average of the observed value
*A*	Scale parameter
*B*	Displacement parameter
*E*	Loss function
*G*	Input activation function
*H*	Output activation function
*P*	Steps forward
*R* and *W*	Earning matrices
R${}^{2}$	Coefficient of determination
α	Learning rate
β	Decay rate
η	Number of predictions
σ	Activation function
ι	Input gate
θ	Vector of parameters
Δ	Period of the samples
Ψ	Mother wavelet
